# Mood, activity, and sleep measured via daily smartphone-based self-monitoring in young patients with newly diagnosed bipolar disorder, their unaffected relatives and healthy control individuals

**DOI:** 10.1007/s00787-020-01611-7

**Published:** 2020-08-02

**Authors:** Sigurd Arne Melbye, Sharleny Stanislaus, Maj Vinberg, Mads Frost, Jakob Eyvind Bardram, Kimie Sletved, Klara Coello, Hanne Lie Kjærstad, Ellen Margrethe Christensen, Maria Faurholt-Jepsen, Lars Vedel Kessing

**Affiliations:** 1 The Copenhagen Affective Disorder Research Center (CADIC), Psychiatric Center Copenhagen, Rigshospitalet, 2100 Copenhagen, Denmark; 2 Faculty of Health and Medical Sciences, University of Copenhagen, Copenhagen, Denmark; 3 Monsenso ApS, Langelinie Allé 47, Copenhagen, Denmark; 4 Psychiatric Research Unit, Psychiatric Centre North Zealand, Hillerød, Denmark; 5 Department of Health Technology, Technical University of Denmark, Kgs. Lyngby, Denmark

**Keywords:** Bipolar disorder, Smartphones, Mood, Activity, Sleep

## Abstract

Diagnostic evaluations and early interventions of patients with bipolar disorder (BD) rely on clinical evaluations. Smartphones have been proposed to facilitate continuous and fine-grained self-monitoring of symptoms. The present study aimed to (1) validate daily smartphone-based self-monitored mood, activity, and sleep, against validated questionnaires and clinical ratings in young patients with newly diagnosed BD, unaffected relatives (UR), and healthy controls persons (HC); (2) investigate differences in daily smartphone-based self-monitored mood, activity, and sleep in young patients with newly diagnosed BD, UR, and HC; (3) investigate associations between self-monitored mood and self-monitored activity and sleep, respectively, in young patients with newly diagnosed BD. 105 young patients with newly diagnosed BD, 24 UR and 77 HC self-monitored 2 to 1077 days (median [IQR] = 65 [17.5–112.5]). There was a statistically significantly negative association between the mood item on Hamilton Depression Rating Scale (HAMD) and smartphone-based self-monitored mood (*B* = − 0.76, 95% CI − 0.91; − 0.63, *p* < 0.001) and between psychomotor item on HAMD and self-monitored activity (*B* = − 0.44, 95% CI − 0.63; − 0.25, *p* < 0.001). Smartphone-based self-monitored mood differed between young patients with newly diagnosed BD and HC (*p* < 0.001), and between UR and HC (*p* = 0.008) and was positively associated with smartphone-based self-reported activity (*p* < 0.001) and sleep duration (*p* < 0.001). The findings support the potential of smartphone-based self-monitoring of mood and activity as part of a biomarker for young patients with BD and UR. Smartphone-based self-monitored mood is better to discriminate between young patients with newly diagnosed BD and HC, and between UR and HC, compared with smartphone-based activity and sleep.

**Trial registration** clinicaltrials.gov NCT0288826

## Background

Bipolar disorder (BD) is a recurrent heterogeneous, and disabling disorder, with a prevalence of 1%, and a heritability of 60–80% [1]. BD is characterized by episodes of mood disturbances with depressive episodes or manic/mixed episodes intervened by euthymic episodes [2]. BD often has an onset of symptoms during young age and may cause substantial functional impairments also before the time of diagnosis, and a progression of severity during years of untreated illness, stressing the need for early diagnosis and intervention [3–5]. An increasing focus has recently been on coexisting alterations in mood, activity and sleep, and loss of function, both during affective episodes, but also between affective episodes [6–8]. Currently, no objective biomarkers like blood tests or radiologic findings are available to diagnose or monitor BD. Thus, symptom monitoring, diagnostic evaluations, and early interventions rely on clinical evaluations typically made with large intervals between outpatient visits, making them vulnerable to recall bias [9] and not capturing daily fluctuations.

Smartphones are owned by 95% of teens in the USA [10], and are always available to most people during naturalistic settings as a natural part of everyday life, especially for young people [11]. Thus, smartphones may facilitate continuous and fine-grained daily self-monitoring of symptoms like mood, activity, and sleep.

Alteration of mood is a central core feature of BD. Many patients with BD experience subsyndromal mood swings on a daily basis that are associated with increased risk of relapse and hospitalization [12–14]. Our group has in recent studies showed that smartphone-based daily self-monitored mood alterations are associated with increased perceived stress and decreased quality of life and functioning [15, 16] and that increased mood instability may behave as a genetic vulnerability trait for BD being present in remitted BD patients [15] and in their unaffected relatives (UR) [16].

Alterations in the level of activity (psychomotor activity) has in the diagnostic and statistical manual of mental disorders (DSM-5) been highlighted as a core symptom similar to mood in BD [17], and elevated activity levels are associated with (hypo)mania, and reduced activity levels are associated with depression [18]. Furthermore, regardless the affective state, patients with newly diagnosed BD have been shown to have lower activity levels compared with healthy individuals [19, 20]. Several studies on adult patients with BD have found smartphone-based self-monitoring of activity feasible [21–23]. A recent study from our group found smartphone-based self-monitored activity to be statistically significantly associated with activity-related sub-items in the Hamilton Depression Rating Scale (HAMD) [24] and the Young Mania Rating Scale (YMRS) [25, 26].

(Hypo)manic episodes are often associated with a substantial reduced sleep duration, while depressive episodes often are associated with sleep disturbances either as a reduced sleep duration or an increased sleep duration [27]. Sleep assessment is often based on prospective reporting in questionnaires or clinical interviews and is, therefore, prone to recall bias [28]. Thus, a tool for a ubiquitous self-monitoring of sleep might eliminate the risk of recall bias. As sleep disturbance is a key symptom in the beginning of, or preceding an affective episode [29], continuous monitoring of sleep can facilitate early identification and intervention of relapsing affective episodes [30].

First-degree relatives to patients with BD have an increased risk of developing psychiatric disorders [31]. Results from a prior study suggest that 20–30% of healthy first-degree relatives of patients with BD will develop affective illness, compared to 2–5% among healthy individuals [32]. Therefore, it is likely that UR to patients with BD will show alterations in mood, activity, and sleep intermediate between young patients with BD and healthy controls (HC), maybe as an expression of prodromal symptoms [32]. Additionally, if smartphone-based self-monitoring is able to identify subsyndromal symptoms in UR, it might have the potential to identify prodromal symptoms in relation to relapse of affective episodes in patients with BD [33], and also identify early symptoms in individuals undergoing diagnostic assessment for BD. Smartphone-based self-monitoring has been tested as a screening-tool for BD in a community sample, with promising results [34]. Also, one study showed that 38% of participants showing positive results in a smartphone-based screening for depression, consulted health care professionals about their results [35].

Smartphone-based self-monitored data have shown to validly reflect illness activity in patients with BD [15, 21, 36–38]. Thus, smartphone-based self-monitoring may improve the timeliness in monitoring symptoms, functioning, and fluctuations in symptoms, and might, therefore, be a promising supportive diagnostic tool in individuals where BD is suspected.

A recent review, conducted by the authors of the present study, showed that smartphone-based self-monitoring in young people with psychiatric disorders generally has both high adherence and acceptability [39]. More specifically, a recent study on electronic ecological momentary assessment in young patients with BD showed adherence rates of 80.4% among young patients with BD [40]. Also, prior research has found smartphone monitoring to be useful, easy to use, and feasible for adult patients with BD [21, 38, 41, 42]. However, no studies on smartphone-based monitoring of young patients with newly diagnosed BD, UR, and HC have been conducted.

## Aims

The present study aimed to (1) validate daily smartphone-based self-monitored mood, activity, and sleep, against validated questionnaires and clinical ratings in young patients with newly diagnosed BD, UR, and HC; (2) investigate differences in daily smartphone-based self-monitored mood, activity, and sleep in young patients with newly diagnosed BD, UR, and HC; (3) investigate associations between self-monitored mood and self-monitored activity and sleep, respectively in young patients with newly diagnosed BD.

We hypothesized that (1) daily smartphone-based self-monitored mood, activity, and sleep would be associated with validated and standardized questionnaires and clinical ratings, (2) daily smartphone-based self-monitored mood, activity, and sleep differs between young patients with newly diagnosed BD compared to UR and HC with UR being an intermediary group; (3) daily smartphone-based self-monitored mood would be positively associated with activity and negatively associated with sleep duration in young patients with newly diagnosed BD.

## Methods

The present study included data collected as part of the Bipolar Illness Onset study (the BIO study) [43], which is an ongoing longitudinal observational study including patients with newly diagnosed BD, their UR and HC [44, 45].

### Study design, participants, and settings

As part of the BIO study, we recruited young patients, from 18 to 25 years old, with newly diagnosed BD, their UR, and HC without a psychiatric family history. In the BIO study, all participants are assessed clinically at baseline and annually, combined with blood tests, and at some visits MRI-scan and cognitive tests in addition to annual visits. The young patients with newly diagnosed BD were recruited from the Affective Disorder Clinic, Rigshospitalet, Copenhagen, Denmark. The Affective Disorders Clinic is a highly specialized clinic offering a 2-year course of treatment to every individual newly diagnosed with BD in the larger region of Copenhagen. We also included some younger patients with newly diagnosed BD, aged 15–17, from the Child and Adolescent Mental Health Center in Copenhagen. For the UR group, we included among the first-degree relatives of the patients included in the study. Inclusion criteria for UR were no history of a psychiatric disorder requiring treatment. HC were recruited among blood donors from the Blood Bank at Rigshospitalet, Copenhagen. Inclusion criteria were no history of a psychiatric disorder requiring treatment, personally or in a first-degree relative, and 25 years old or younger at the time of inclusion. All participants signed an informed consent form before starting in the project; also for participants under the age of 18, we obtained informed consent from one or both legal parents.

### Diagnostic assessment

To ensure that participants fulfilled the inclusion criteria to participate in the designated groups, all participants underwent a diagnostic interview using Schedules for Clinical Assessment in Neuropsychiatry (SCAN) [46], during the baseline interview. Trained researchers performed the SCAN interviews; for the BD and UR groups, baseline interviews were performed by Ph.D students in medicine or psychology, whereas the HC were either examined by the same Ph.D students, or medical or psychology students.

### Baseline interview and follow-up

All participants attended a baseline interview with collection of general information about work status, educational status, in addition to diagnostic and clinical assessment. For young patients with newly diagnosed BD, we collected information about time for onset of symptoms, diagnosis, start of treatment, and number and duration of affective episodes. All participants also completed a number of questionnaires. After baseline, participants were asked to attend an annual follow-up interview for a maximum of 5 years. For young patients with newly diagnosed BD, we endeavored to arrange interviews with the participants each time they had a change from one affective episode to another, from an affective episode to euthymic stage, or from a euthymic stage to an affective episode.

### Clinical ratings

At baseline and follow-up, the following clinical assessments were conducted: a clinical assessment of the severity of depressive symptoms was performed using the Hamilton Depression Rating Scale 17-item (HAMD) [24, 47], and of (hypo-)manic symptoms using the Young Mania Rating Scale (YMRS) [26]. From the HAMD, we included the total scores from HAMD 17-item and 6-item as well as the scores on sub-item 1 addressing level of decreased mood, sub-item 4–6 addressing sleep disturbances, sub-item 8 addressing the level of psychomotor retardation, and sub-item 9 addressing the level of psychomotor agitation, for the past 3 days. From the YMRS, we included the total score as well as the scores from sub-item 1 addressing the level of elevated mood, sub-item 2 addressing the level of increased motor activity, sub-item 4 reflecting reduced sleep duration, and sub-item 6 addressing increased talkativeness, for the past 3 days.

Further, a clinical assessment of functioning was performed using the Functioning Assessment Short Test (FAST) [48], which is a test developed explicitly for BD and addresses six areas of functioning: autonomy, occupational functioning, cognitive functioning, financial issues, interpersonal relationship, and leisure time. All 24 items are rated from 0 (no difficulties) to 3 (severe difficulties) and assess the last 2 weeks up to the rating. FAST has a high test–retest reliability and has been validated against the Global assessment of functioning scale (GAF) [48].

### Questionnaires

All participants completed several questionnaires at baseline and follow-up. The questionnaires used for analyses in this article were the following: severity of self-assessed depressive symptoms according to the Major Depression Inventory (MDI) with scores on the MDI ranging from 0 to 50, where a score below 20 indicates no depression and a score over 29 indicates severe depression [49]; severity of self-assessed manic symptoms according to the Altman Self-Rating Mania Scale (ASRM), with scores on the ASRM ranging from 0 to 20, where a score of 6 or higher indicates a high probability of hypomanic or manic state [50]; self-assessed physical activity according to the International Physical Activity Questionnaire (IPAQ), where participants report how many minutes of physical activity in different intensities they had the prior week, these are then converted to metabolic equivalent task (MET) minutes per week, which then adds up to the total MET score [51]; and self-assessed sleep quality according to the Pittsburgh Sleep Quality Index (PSQI), with scores on the PSQI ranging from 0 to 21, where a score over 5 indicates sleep disturbances [52]. These questionnaires were chosen as they reflect the level of mood alteration, activity and sleep quality for the participants. We did not include other questionnaires in the analyses, and all hypotheses and analyses were specified in advance.

### Smartphone-based self-monitoring

The Monsenso system is a smartphone-based self-monitoring system developed by the authors allowing for daily self-monitoring of mood, sleep, and activity [53, 54]. The Monsenso application also collect automatically generated data on phone usage, social activity (usage of calls and text messages) and physical activity; however, only self-monitored data are investigated in this article. The system is available for both Android and iOS; however, collection of automatically generated data is only for Android phones. These automatically generated data were the only ones which were collected due to technical practicalities.

At the time of inclusion, every participant was registered in the Monsenso system and instructed to start using the system 3 days before the baseline interview. Participants installed the app on their phone, and participants not owning a phone compatible with Monsenso were offered to borrow an Android smartphone (LG Nexus 5). Young patients with newly diagnosed BD were asked to use the system for at least 3 months, and the remaining participants were asked to use the system for at least 1 month. Participants were not instructed on what time of the day to perform the self-monitoring. When participants were booked for follow-up interviews with clinical assessments, they were encouraged to start using the system again.

**Fig. 1:**
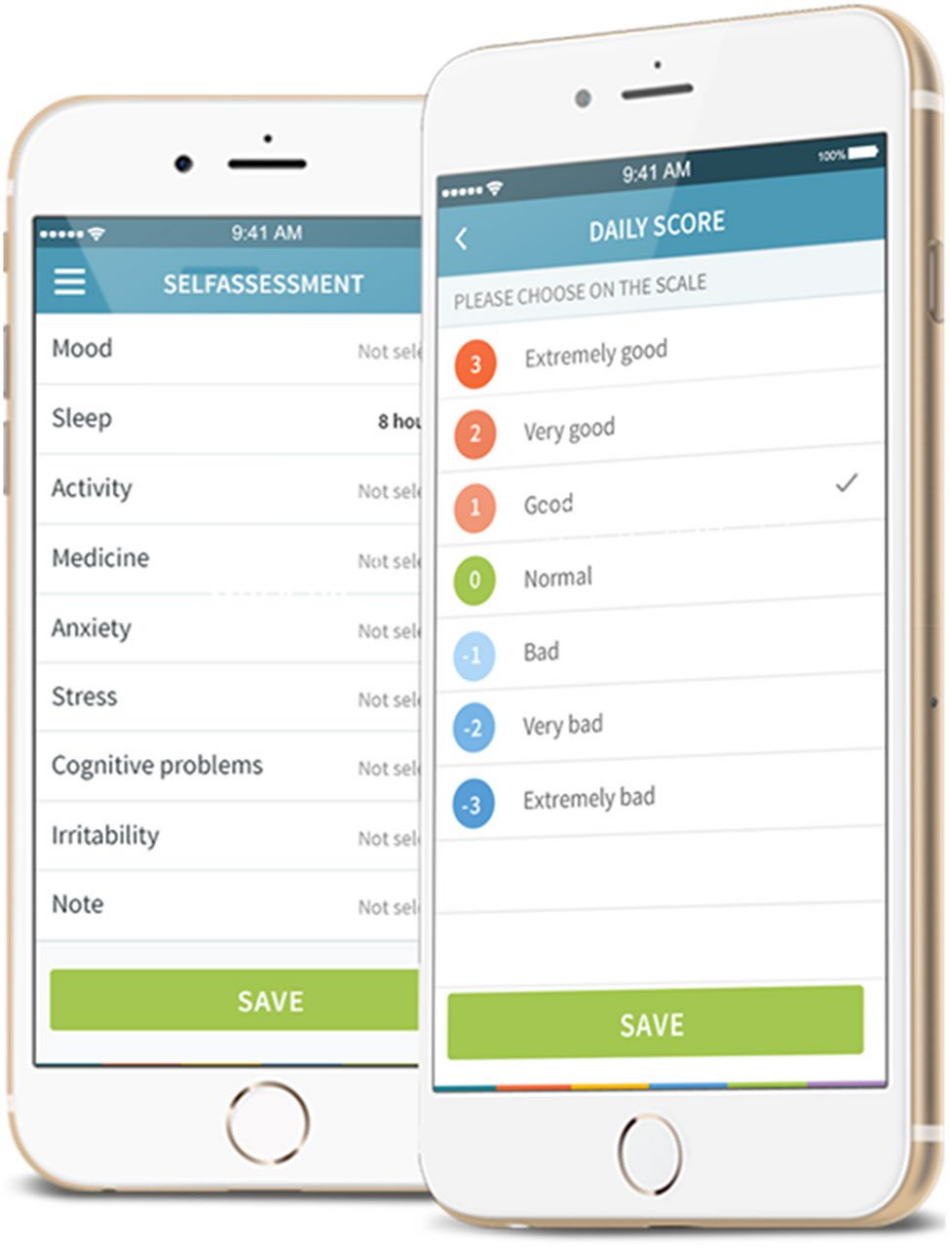
Screenshots from the Monsenso interface

When installed on the phone, the application prompted the user to fill out the self-monitoring once a day. As default, prompts were set for 8 pm daily, but participants could set their preferred time for prompts, or deactivate prompts completely. When prompted (or reminded by memory), the participant opened the application and completed as many features of the self-assessment as wanted. Figure 1 shows screenshots of the interface. The self-monitoring took 2–4 min each day.

For self-monitoring of mood, young patients with newly diagnosed BD, UR and HC scored how their days had been on a 7-point-scale (− 3, − 2, − 1, 0, 1, 2, 3). In this way, all participants daily scored how good or how bad their day had been. Additionally, young patients with newly diagnosed BD scored their mood on a 9-point scale with scores from depressive to manic (− 3, − 2, − 1, − 0.5, 0, 0.5, 1, 2, 3), as a more fine-grained scale has been found useful for individuals with BD [53]. For self-assessment of activity, participants scored their daily level of activity on a 7-point-scale (− 3, − 2, − 1, 0, 1, 2, 3). All participants also registered their bedtime and the time they got out of bed. Self-monitoring of mood, activity and sleep were part of the default interface in the application, and the only features included in the analyses; however, it was possible to add other prefabricated features, or even create custom made features.

### Statistical analyses

The hypotheses and statistical analyses for the present study were defined a priori. For background characteristics, linear mixed effect models were used for analyzing the differences in the mean of continuous variables between the three groups. For between-group differences of categorical data, Chi-square tests were conducted. Concerning analyses for aims 1–3, for each measure of interest, a linear mixed effect model, which accommodates both the variation of the variables of interest within young patients with newly diagnosed BD (intra-individual variation) and between individuals (inter-individual variation) was employed. Because the data used in this article are part of a larger still ongoing longitudinal study [43], we had data from both baseline and, for some participants, up to a maximum of nine follow-ups. Thus, we used a linear mixed effect model analysis to account for repeated measurements within each participant. As mixed effect models are more robust in dealing with missing data in repeated measurements, we could include all data from all of our participants, regardless of large differences in the amount of data from each participant. We identified all participants with a unique identification (ID) number and a family number. Each patient was given a family number, and if they had a relative in the study, they were given the same family number as the patient. Thus, the familial relationship was accounted for by adding family number as a random factor in the linear mixed effect model. For aim (1), we used a linear mixed effect model to analyze the association between smartphone-based self-assessed scores and scores from clinical rating scales and questionnaires. For analyses on associations between smartphone-based monitoring and clinical ratings and questionnaires averages of smartphone-based score ratings were calculated for the current day and 3 days before ratings with the HAMD and the YMRS, 7 days prior for the ASRM, and 14 days prior for the MDI and the FAST, and 30 days prior for the PSQI. For aim (2), we used a linear mixed effect models to compare means between smartphone-based self-assessments scores, scores from clinical ratings, and scores from questionnaires.

For aim (3), we used a linear mixed effect model to analyze the associations between smartphone-based self-assessed mood and smartphone-based self-assessed activity and sleep, respectively. For all analyses, we added individual ID number and family number as random factors.

For aim (1) and (2), we analyzed pooled data from all participants; for aim (3), we analyzed data on young patients with newly diagnosed, only. Analyses were conducted in models adjusted for age and sex.

As few prior studies have investigated differences in daily smartphone-based self-monitored symptoms between young patients with newly diagnosed BD, UR, and HC, statistical power analyses prior to the study were not performed. Data were collected as part of a larger longitudinal observational study, and thus the sample size was defined according to this. Model assumptions were checked visually by means of residuals and QQ plots for each of the statistical analyses. We used SPSS version 25 (Statistical Package for the Social Sciences) for all analyses. *p* values ≤ 0.05 (two sided) were considered statistically significant.

## Results

### Demographic and clinical characteristics

A total of 401 young patients with newly diagnosed BD, 92 UR, and 200 HC from September 2016 to November 2019 were included in the BIO study. Of these, a total of 105 young patients with newly diagnosed BD, 24 UR, and 77 HC provided smartphone-based self-monitoring data and were 25 years old or younger by the time of inclusion and thus eligible to be included in the analyses for the present study. There were 3 pairs of relatives among the 23 UR. The rate of participants taking part of the study after talking to one of the researchers was very high (< 85%); however, these were already screened by clinicians regarding interest in participating. Further background characteristics are presented in Table 1.

**Table 1:** Demographics and background characteristics of young patients with newly diagnosed bipolar disorder (BD), unaffected relatives (UR), and healthy control individuals (HC); *N* = 206

	BD	UR	HC	BD vs UR	BD vs HC	UR vs HC
				*p*	*p*	*p*
Participants	105	24	77			
Age, years	21.74 (21.28; 22.21)	23.08 (21.40; 24.77)	22.87 (22.45; 23.29)	*0.012*	*0.002*	0.755
Female, % (*n*)	75.2 (79)	60.9 (14)	76.6 (59)	0.205	0.829	0.172
Education, years	13 [11.5–14.5]	15 [13–17]	15 [11.5–14.5]	*0.004*	*< 0.001*	0.404
Student, % (*n*)	60.9 (64)	50 (12)	61 (47)	0.325	0.991	0.338
Full time employment, % (*n*)	12.4 (13)	37.5 (9)	13 (10)	*0.003*	0.903	*0.007*
HAMD-17	8 [2.5–13.5]	1.5 [0–5.5]	0 [0–2]	*< 0.001*	*< 0.001*	0.107
YMRS	3 [0–7.5]	0 [0–2]	0 [0–1]	*< 0.001*	*< 0.001*	0.374
FAST, total score	19 [8.5–29.5]	2 [0–5]	1 [0–2]	*< 0.001*	*< 0.001*	0.079
Bipolar disorder subtype II, % (*n*)	72.4 (76)	–	–	–	–	–
Age of illness onset, years	15 [13–17]	–	–	–	–	–
Illness duration, years^a^	6 [3–9]	–	–	–	–	–
Number of years untreated^b^	3 [0.5–5.5]	–	–	–	–	–
No. of depressive episodes	5 [1.5–8.5]	–	–	–	–	–
No. of hypomanic episodes	4 [0–8]	–	–	–	–	–
No. of manic episodes	0 [0–1]	–	–	–	–	–
No. of mixed episodes	0 [0–0]	–	–	–	–	–
No. of total episodes	12 [5.5–18.5]	–	–	–	–	–

Continuous variables are presented as median [interquartile range] or mean (SD) and *p* values are calculated based on differences in the mean between the three groups using linear mixed models. Categorical data are presented as % (*n*), and *p* values are calculated using the Chi-square test

HAMD-17: Hamilton Depression Rating Scale with 17 items version; YMRS: Young Mania Rating Scale; FAST: Functional Assessment Short Test

*p* values < 0.05 are presented in italic to emphasize statistical significance

^a^llIness duration was defined as the time from the first episode to the time of inclusion

^b^Untreated BD was defined as the time from the first mania, hypomania, or mixed episode to time of diagnosis

### Follow-up and smartphone-based self-assessment

**Fig. 2:**
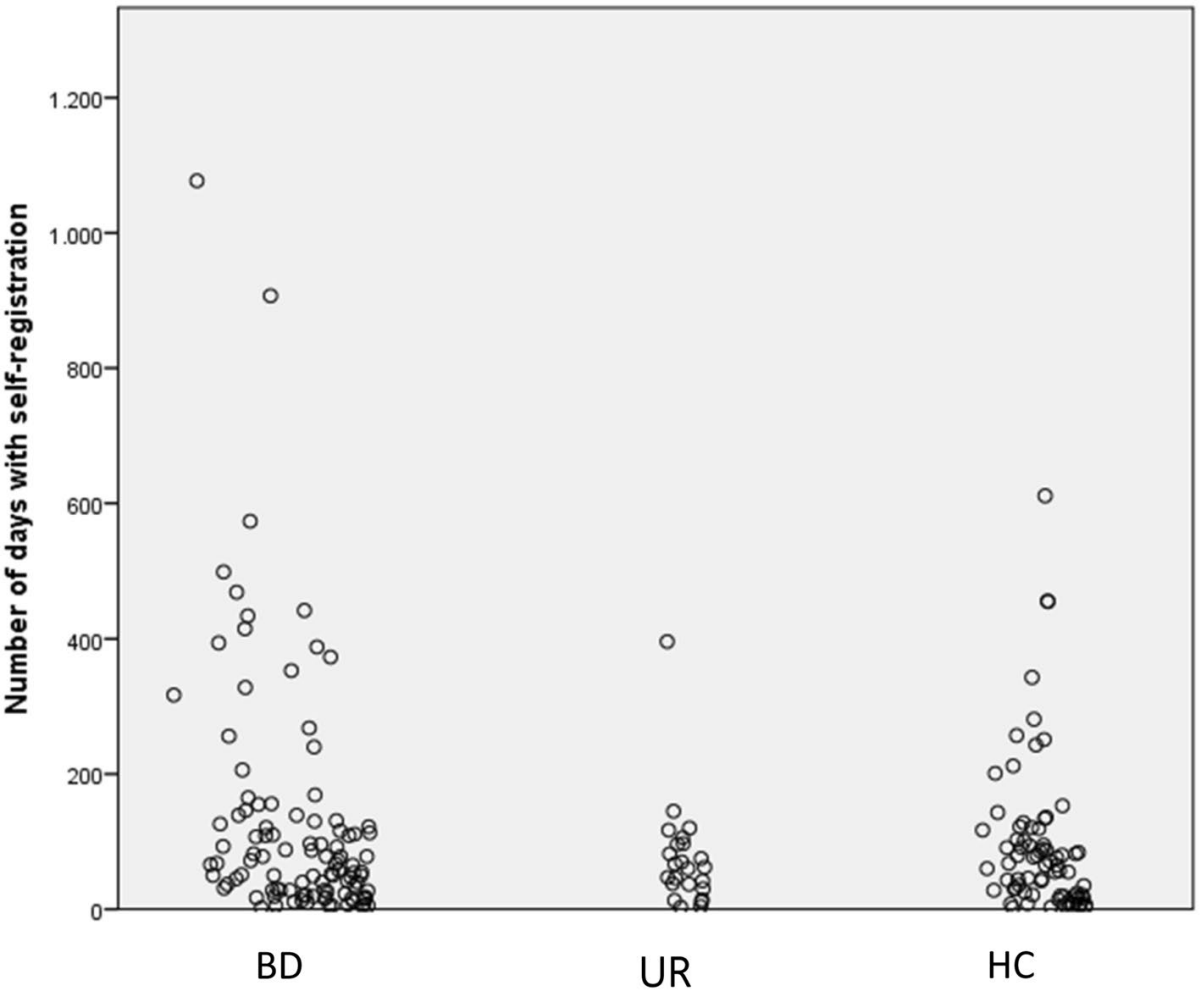
The number of days with smartphone-based self-monitoring in young patients with newly diagnosed bipolar disorder (BD), unaffected relatives (UR), and healthy control individuals (HC). Each circle represents a participant

There were a total number of 342 visits with clinical assessment among the participants, and for 262 of these visits, the participants provided daily smartphone-based self-assessment. The number of visits for each participant varied from one to nine visits. The number of days where participants performed self-assessment varied from 2 to 1077 days (median [IQR] = 65 [17.5–112.5]) (Fig. 2).

### The validity of smartphone-based self-monitored mood, activity, and sleep compared with clinical ratings and questionnaires

#### Mood

As shown in Table 2, there was a statistically significant negative association between self-monitored mood and scores on subitem 1 of the HAMD (*B *=− 0.76, 95% CI − 0.90; − 0.63, *p *< 0.001); as well as the total score of HAMD-17 (*B *=− 0.11, 95% CI − 0.12; − 0.09, *p *< 0.001) and HAMD-6 rating scale (*B *=− 0.18, 95% CI − 0.21; − 0.15, *p *< 0.001). Thus, for every decrease of 1 point on the HAMD sub-item 1 score, there was a 0.76 increase on the smartphone-based self-monitoring of mood. There was no statistically significant association between self-monitored mood and the YMRS, neither for subitem 1 or total score. For questionnaires, there was a statistically significant negative association between smartphone-based self-monitored mood and scores on the MDI (*B *=− 0.05, 95% CI − 0.06; − 0.04, *p *< 0.001), and a statistically significant positive association between smartphone-based self-monitored mood and scores on the ASRM (*B *=0.06, 95% CI 0.02; 0.10, *p* = 0.001). Thus, for every decrease of 1 point on the MDI total score, there was a 0.05 increase on the smartphone-based self-monitoring of mood. For every increase of 1 point on the ASRM score, there was a 0.06 increase on the smartphone-based self-monitoring of mood.

**Table 2:** Associations between smartphone-based self-monitored mood, activity and sleep (dependent variables), and observer-based rating scales for depression, mania, functioning, and questionnaires for sleep and physical activity (data pooled for young patients with newly diagnosed bipolar disorder, unaffected relatives, and healthy control individuals), *N* = 190

	*B*	95% CI	*p*
Smartphone-based self-monitored mood			
HAMD-17 total^a^	− 0.11	− 0.12; − 0.09	*< 0.001*
HAMD-6 total^b^	− 0.18	− 0.21; − 0.15	*< 0.001*
HAMD sub-item 1^c^	− 0.76	− 0.90; − 0.63	*< 0.001*
YMRS total^d^	− 0.02	− 0.05; 0.02	0.277
YMRS sub-item 1^e^	0.16	− 0.04; 0.35	0.112
FAST^f^	− 0.05	− 0.05; − 0.04	*< 0.001*
MDI^g^	− 0.05	− 0.06; − 0.04	*< 0.001*
ASRM^h^	0.06	0.02; 0.10	*0.001*
Smartphone-based self-monitored activity			
HAMD-17 sub-item 8^i^	− 0.44	− 0.63; − 0.25	*<0.001*
HAMD-17 sub-item 9^j^	− 0.37	− 0.58; − 0.15	*0.001*
YMRS sub-item 2^k^	0.21	0.02; 0.39	*0.028*
YMRS sub-item 6^l^	0.05	− 0.08; 0.19	0.429
FAST^f^	− 0.02	− 0.03; − 0.01	*< 0.001*
IPAQ^m^	3.94E−5	1.09E−5; 6.79E−5	*0.007*
Smartphone-based self-monitored sleep duration			
HAMD-17 sub-item 4^n^	− 8.72	− 18.365; 0.917	0.076
HAMD-17 sub-item 5^o^	− 3.37	− 13.616; 6.867	0.517
HAMD-17 sub-item 6^p^	− 0.89	− 13.035; 11.260	0.886
YMRS sub-item 4^q^	− 29.65	− 43.514; − 15.793	*< 0.001*
FAST^f^	0.377	− 0.195; 0.949	0.196
PSQI^r^	− 2.94	− 5.065; − 0.819	*0.007*

Model adjusted for age and gender

*p* values < 0.05 are presented in italic to emphasize statistical significance

^a^HAMD-17: The Hamilton Depression Rating Scale 17 total score

^b^HAMD-6 Hamilton depression Rating scale 6 total score

^c^HAMD sub-item 1—level of decreased mood

^d^YMRS: The Young Mania Rating Scale total score

^e^YMRS sub-item 1—level of elevated mood

^f^FAST: The Functional Assessment Short Test total score

^g^MDI: The Major depression Inventory total score

^h^ASRM: The Altman Self-Rating Mania Scale total score

^i^HAMD sub-item 8—level of psychomotor retardation

^j^HAMD sub-item 9—level of psychomotor agitation

^k^YMRS sub-item 2—level of increased motor activity

^l^YMRS sub-item 6—increased talkativeness

^m^IPAQ: The Physical Activity Questionnaire—short form total score

^n^HAMD sub-item 4—problems falling asleep

^o^HAMD sub-item 5—problems with mid-sleep–wake-ups

^p^HAMD sub-item 6—problems with early morning awakening

^q^YMRS Sub-item 4—reduced sleep duration

^r^PSQI: The Pittsburgh Sleep Quality Index total score

#### Activity

There was a statistically significantly negatively association between smartphone-based self-monitored activity and the HAMD subitem 8 (*B *=− 0.44, 95% CI − 0.63; − 0.25, *p* < 0.001) and 9 (*B *=− 0.37, 95% CI − 0.58; − 0.15, *p* = 0.001), and total scores on the FAST (*B *=− 0.02, 95% CI − 0.03; − 0.01, *p *< 0.001). Thus, for every increase of 1 point on the HAMD sub-item 8 score, there was a 0.44 decrease on the smartphone-based self-monitoring of activity, for every increase of 1 point on the HAMD sub-item 9 score, there was a 0.37 decrease on the smartphone-based self-monitoring of activity, and for every 1 point increase on the FAST total score, there was a 0.02 decrease on the smartphone-based self-monitoring of activity. Smartphone-based self-monitored activity was significantly positively associated with the YMRS subitem 2 (*B *=0.21, 95% CI 0.02; − 0.39, *p* = 0.028). Thus, for every increase of 1 point on the YMRS sub-item 2 score, there was a 0.21 increase on the smartphone-based self-monitoring of activity. There was no statistically significant association between smartphone-based self-monitored activity and the YMRS subitem 6. There was a statistically significant positive association between self-monitored activity and total score for the IPAQ (*B *=3.94E−5, 95% CI 1.09E−5; 6.79E−5, *p* = 0.007).

#### Sleep

There was no statistically significant association between smartphone-based self-monitored sleep duration and scores on the HAMD subitems 4, 5, or 6. Nevertheless, there was a statistically significant negative association between smartphone-based self-monitored sleep duration and the YMRS subitem 4 (*B *=− 29.65, 95% CI − 43.51; − 15.80, *p* < 0.001), as well as between smartphone-based self-monitored sleep duration and total scores on the PSQI (*B *=− 2.94, 95% CI − 5.07; − 0.82, *p* = 0.007). Thus, for every increase of 1 point on the YMRS sub-item 4 score, there was a 29.65 min decrease on the smartphone-based self-monitoring of sleep duration, and for every increase of 1 on the PSQI total score, there was a 2.94 min increase on the smartphone-based self-monitoring of sleep duration.

### Differences in daily smartphone-based self-monitoring of mood, activity, and sleep between young patients with newly diagnosed bipolar disorder, unaffected relatives and healthy controls

Table 3 shows the differences in means of scores of smartphone-based self-monitored mood, activity and sleep, and some of the clinical ratings between young patients with newly diagnosed BD, UR and HC. There was no statistically significant difference in smartphone-based self-monitored mood between young patients with newly diagnosed BD and UR (*p *= 0.107). There was a statistically significant difference between young patients with newly diagnosed BD and HC (*p *< 0.001), and between UR and HC (*p *= 0.008). For smartphone-based self-monitored activity, there was no statistically significant difference between any of the groups. For smartphone-based self-monitored sleep duration, there was a statistically significant difference between young patients with newly diagnosed BD and HC (*p *= 0.003), and no other statistically significant between-group difference.

**Table 3:** Estimated differences in mood, activity, and sleep indexes between young patients with newly diagnosed bipolar disorder (BD, *n* = 105), unaffected relatives (UR, *n* = 24) and control individuals (HC, *n* = 77), *N* = 206

	BD		UR		HC		BD/UR	BD/HC	UR/HC
	Mean	95% CI	Mean	95% CI	Mean	95% CI	*p*	*p*	*P*
Smartphone-based self-monitored mood									
Mood^a^	0.41	0.15; 0.68	0.89	0.35; 1.44	1.71	1.42; 1.10	0.107	*< 0.001*	*0.008*
Mood measures in HAMD and YMRS									
HAMD subitem 1^b^	0.63	0.51; 0.74	0.06	− 0.17; 0.28	0.06	− 0.07; 0.20	*< 0.001*	*< 0.001*	0.982
YMRS subitem 1^c^	0.55	0.44; 0.66	0.17	− 0.05; 0.39	0.22	0.09; 0.35	*0.003*	*< 0.001*	0.694
Total score in MDI and ASRM									
MDI^d^	20.28	18.59; 21.97	9.76	6.54; 12.97	5.50	3.55; 7.45	*< 0.001*	*< 0.001*	*0.023*
ASRM^e^	4.13	3.50; 4.75	2.61	1.43; 3.80	2.94	2.23; 3.66	*0.024*	*0.010*	0.631
Smartphone-based self-monitored activity									
Activity score^f^	0.41	0.14; 0.67	0.76	0.24; 1.27	0.70	0.41; 0.98	0.226	0.124	0.823
Activity measures on HAMD and YMRS									
HAMD sub-item 8^g^	0.35	0.26; 0.43	0.01	− 0.15; 0.18	0.00	− 0.10; 0.10	*< 0.001*	*< 0.001*	0.894
HAMD sub-item 9^h^	0.34	0.26; 0.42	0.17	0.01; 0.32	0.02	− 0.07; 0.11	*0.048*	*< 0.001*	0.111
YMRS sub-item 2^i^	0.48	0.36; 0.60	0.10	− 0.14; 0.33	0.11	− 0.03; 0.25	*0.004*	*< 0.001*	0.903
YMRS sub-item 6^j^	0.78	0.63; 0.94	0.04	− 0.27; 0.33	0.10	− 0.08; 0.28	*< 0.001*	*< 0.001*	0.697
International Physical Activity Questionnaire									
FAST total score^k^	18.02	16.30;19.73	3.55	0.18; 6.93	1.12	− 0.85; 3.09	*< 0.001*	*< 0.001*	0.209
Functional Assessment Short Test									
IPAQ total score^l^	3416	2664; 4168	2725	1381; 4069	3415	2593; 4236	0.356	0.997	0.377
Smartphone-based self-monitored sleep									
Sleep duration (hours:min)^m^	6:48	6:27; 7:09	7:04	6:23; 7:44	7:31	7:09; 7:54	0.489	*0.003*	0.225
Sleep measures on HAMD^f^ and YMRS									
HAMD sub-item 4–6^n^	2.45	2.18; 2.72	1.03	0.51; 1.55	0.69	0.38; 0.10	*< 0.001*	*< 0.001*	0.251
YMRS sub-item 4^o^	0.46	0.35; 0.58	0.11	− 0.11; 0.33	0.08	− 0.05; 0.21	*0.005*	*< 0.001*	0.805
Pittsburgh sleep Quality Index									
Total PSQI score^p^	8.66	8.09; 9.22	5.42	4.41; 6.43	4.38	3.76; 4.99	*< 0.001*	*< 0.001*	0.075

Adjusted for age and sex

*p* values < 0.05 are presented in italic to emphasize statistical significance

^a^Smartphone-based self-monitored mood: Averages of smartphone-based day score

^b^HAMD sub-item 1—level of decreased mood

^c^YMRS sub-item 1—level of elevated mood

^d^MDI: The Major depression Inventory total score

^e^ASRM: The Altman Self-Rating Mania Scale total score

^f^Smartphone-based self-monitored activity: averages of smartphone-based activity rating

^g^HAMD sub-item 8—level of psychomotor retardation

^h^HAMD sub-item 9—level of psychomotor agitation

^i^YMRS sub-item 2—level of increased motor activity

^j^YMRS sub-item 6—increased talkativeness

^k^FAST: The Functional Assessment Short Test total score

^l^IPAQ: The Physical Activity Questionnaire—short form total score

^m^Smartphone-based self-monitored sleep duration: Averages of smartphone-based sleep length (minutes from bedtime to time they got out of bed)

^n^HAMD sub-item 4–6—sum of scores regarding problems with sleep

^o^YMRS sub-item 4—reduced amount of sleep last 3 days

^p^PSQI: The Pittsburgh Sleep Quality Index total score

There were, however, statistically significant differences between groups with clinical ratings and questionnaires, mainly between BD and UR and between BD and HC.

### Association between smartphone-based self-monitored mood, activity, and sleep

Table 4 shows the associations between smartphone-based self-monitored mood, activity and sleep duration, respectively, in young patients with newly diagnosed BD. Analyses both with 7-point mood scale and the finer grained 9-point mood scale (both ranging from − 3 to 3) were employed. There was a statistically significant positive association between smartphone-based self-monitored mood and activity for both the 7-point (*B *= 0.37, 95% CI 0.36; 0.39, *p* < 0.001) scale and 9-point scale (*B *=0.16, 95% CI 0.15; 0.17, *p* = < 0.001). Thus, for every increase of 1 point on the smartphone-based self-monitoring of activity, there was a 0.37 increase on self-reported mood on the 7-point scale, and a 0.16 increase on self-reported mood on the 9-point scale. There was a statistically significant negative association between smartphone-based self-monitored mood and self-monitored sleep duration for the 9-point mood scale (*B *=− 0.0007, 95% CI − 0.0008; − 0.0006, *p* < 0.001), and for the 7-point mood scale (*B *=− 0.0007, 95% CI − 0.0009; − 0.0005 *p* < 0.001). Thus, for every increase of 1 min on self-reported sleep duration, there was 0.0007 decrease on smartphone-based self-monitoring of mood.

**Table 4:** Associations between smartphone-based self-monitored mood (dependent variable), and smartphone-based self-monitored sleep and activity, respectively, for young patients with newly diagnosed bipolar disorder (*N* = 105)

	*B*	95% CI	*p*
Smartphone-based self-monitored mood (7-point scale)^a^			
Smartphone-based self-monitored activity^b^	0.37	0.36; 0.39	*< 0.001*
Smartphone-based self-monitored sleep duration^c^	− 0.0007	− 0.0009; − 0.0005	*< 0.001*
Smartphone-based self-monitored mood (9-point scale)^d^			
Smartphone-based self-monitored activity	0.16	0.15; 0.17	*< 0.001*
Smartphone-based self-monitored sleep duration	− 0.0007	− 0.0008; − 0.0006	*< 0.001*

Adjusted for age and gender

*p* values < 0.05 are presented in italic to emphasize statistical significance

^a^Smartphone-based self-monitored mood rated on a 7-point scale from − 3 to + 3

^b^Smartphone-based self-monitored activity rated on a 7-point scale from − 3 to + 3

^c^Smartphone-based self-monitored sleep duration reported in minutes from bedtime to the time they got out of bed

^d^Smartphone-based self-monitored mood rated on a 9-point scale from − 3 to + 3

## Discussion

This observational study is the first to investigate smartphone self-monitoring of mood, activity, and sleep duration in young patients with newly diagnosed BD, UR and HC. The study included 206 participants who used the Monsenso smartphone application for self-monitoring for up to 3 years, in addition to multiple clinical assessments.

Our results suggest that smartphone-based self-monitoring is a valid presentation of mood in young patients with depressive symptoms, and self-monitored activity gives a valid presentation of the level of activity detected in clinical ratings.

Aim 1: In accordance with our hypotheses, we found a statistically significant association between smartphone-based self-monitored mood and scores on the mood item 1 and the total HAMD. This finding is in line with findings from similar studies on adults with BD [15, 21, 36, 37], and indicates that smartphone-based self-monitoring can identify depressive symptoms and thus function as a fine-grained monitoring tool for young patients with BD, and as a potential diagnostic tool for patients where BD or unipolar disorder is suspected. There was no significant association between self-monitored mood and scores on the YMRS. This may be due to a low number of observations of young patients with newly diagnosed BD clinically assessed during a manic episode, and due to UR and HC rating high on self-reported mood, without having manic symptoms. Thus, the latter results should be interpreted with caution.

Also, in accordance with our hypotheses, self-monitored activity was associated with the HAMD subitems concerning activity and with one of two activity related YMRS items, showing that this monitoring feature also may serve as a monitoring tool for young patients with BD. There was a significant association between self-monitored sleep duration and the sleep-related YMRS subitem; however, no significant correlations between self-monitored sleep duration and sleep-related HAMD subitems. A reason for this can be that depression is associated with both increased and decreased sleep duration, and that self-monitoring of sleep needs more detailed features like e.g., number of times woken up, quality of sleep etc.

Aim 2: For self-monitored mood, there was a statistically significant difference both between UR and HC, and between young patients with newly diagnosed BD and HC, which indicates that UR can have intermediary symptoms. Nevertheless, there was no significant difference between young patients with newly diagnosed BD and UR. The overall findings suggests that the self-monitoring can be used to identify subsyndromal symptoms, which can be very useful as a diagnostic tool and in early intervention, as it potentially can identify prodromal symptoms which sometimes patients with BD have difficulties identifying themselves [33].

Aim 3: The significant positive correlation between self-monitored mood and self-monitored activity and between self-monitored mood and self-monitored sleep duration in young patients with newly diagnosed BD, supports the clinically known associations between mood, and activity and sleep, respectively, and thus suggest that these features may constitute a part of an electronic biomarker, as it also gives more insight to the patients complex distribution of symptoms, potentially identifying therapeutic targets [55].

## Limitations

First, in addition to the smartphone-based self-monitoring data, participants in the present study provided blood samples, cognitive tests, and MRI as part of the BIO study, making smartphone-based self-monitoring an additional part of the study participation. This might have resulted in a lower adherence, compared to if the study focused on smartphone-based monitoring only. Thus, we saw a big variety in adherence reflecting the difference in the participant’s dedication for the smartphone-based part of the BIO study. Second, the number of UR in this study was rather small, partly due to the criteria of no prior history of mental disorders and partly due to patients with BD not approving us to contact their relatives. Third, it was challenging to get the young patients with newly diagnosed BD to revisit for clinical assessment during more severe affective episodes. The Monsenso system, also collects automatically generated data which gives an even more fine-grained and objective information regarding activity and sleep; however, this is only available for Android phones. As the share of Android users among our participants in the present study was low, the number of UR contributing with automatically generated data was very low to include these data in the analysis; also, the focus of this article was on self-monitored data.

Our population consisted of 74% female, and among the young patients with newly diagnosed BD, 72% had BD type 2. However, we believe that our results are generalizable to young patients with newly diagnosed BD in general. Lastly, our HC were recruited among blood donors, which might represent a ‘super healthy’ population [17], although other and more suited control groups are difficult to identify and recruit.

### Strengths

The present study systematically recruited 206 young people comprising young patients with newly diagnosed BD, their UR, and HC. Also, the young patients with newly diagnosed BD were diagnosed at a mood disorder clinic and the diagnosis, or lack of diagnoses were verified for all participants with a SCAN-interview conducted by trained MD and M.Sc. psychology Ph.D student assessors. Furthermore, participants were clinically assessed using clinically validated observer-based rating scales like the HAMD, the YMRS, and the FAST. The smartphone-based self-monitoring system (the Monsenso system) used in the present study is well validated, useful and fulfills safety of data storage and privacy requirements.

### Perspectives

The emerging research on smartphone-based monitoring of psychiatric symptoms in young people, has shown this technology to be both feasible and acceptable [39], and our findings suggest that it also produce valid clinical information in young patients with BD and potentially UR. Future studies should investigate the use of automatically generated data, which supplements with more continuous, fine-grained, objective, and real-time clinical information. This provides unique possibilities for a fine-grained real-time monitoring tool that can give clinicians an extended insight into the symptoms of patients and UR and level of function over time and facilitate targeted intervention. Additionally, valid identification of symptoms may improve diagnosing and may result in patients getting the correct diagnoses with less latency.

## Conclusions

Smartphone-based self-monitoring of mood and activity represents valid measurements for young patients with BD and their UR, specifically with depressive symptoms according to validated clinical ratings and self-reported questionnaires. Smartphone-based self-monitored mood was, compared with self-reported activity and sleep, better at discriminating between young patients with newly diagnosed BD and HC, and between UR and HC, with patients scoring lowest, and UR intermediary lower mood scores. Moreover, self-monitored mood correlates with self-reported activity and sleep duration. Overall, the study shows the potential of using smartphone-based self-monitoring as part of an electronic biomarker for BD, as well as for investigating the impact on UR. The findings indicate that UR have a symptom burden intermediary between young patients with newly diagnosed BD and HC regarding mood, whereas activity and sleep do not differ between UR and HC.

## Data Availability

The study is ongoing; therefore, the research data are not shared.
